# The value of anterior displacement of the abdominal aorta in diagnosing neuroblastoma in children*

**DOI:** 10.1590/0100-3984.2015.0138

**Published:** 2016

**Authors:** Jose Luiz de Oliveira Schiavon, Eliana Maria Monteiro Caran, Vicente Odone Filho, Henrique Manoel Lederman

**Affiliations:** 1 Radiologist, Master Student in Science in the Department of Diagnostic Imaging of the Escola Paulista de Medicina da Universidade Federal de São Paulo (EPM-Unifesp), São Paulo, SP, Brazil.; 2 PhD, Pediatric Oncologist, Advisor for the Graduate Program in Pediatrics and Applied Sciences in Pediatrics at the Escola Paulista de Medicina da Universidade Federal de São Paulo (EPM-Unifesp), São Paulo, SP, Brazil.; 3 Oncologist, Tenured Full Professor in the Pediatrics Department of the Faculdade de Medicina da Universidade de São Paulo (FMUSP), São Paulo, SP, Brazil.; 4 Radiologist, Tenured Full Professor and Coordinator of the Graduate Program in Radiological Sciences in the Department of Diagnostic Imaging of the Escola Paulista de Medicina da Universidade Federal de São Paulo (EPM-Unifesp), São Paulo, SP, Brazil.

**Keywords:** Tomography, X-ray computed, Magnetic resonance imaging, Neuroblastoma, Aorta, abdominal, Pediatrics

## Abstract

**Objective:**

To determine the value of anterior displacement of the abdominal aorta, when present at any level or only at the level of the adrenal gland, contralateral to the mass, in diagnosing neuroblastoma on computed tomography or magnetic resonance imaging in children up to 7 years of age.

**Materials and Methods:**

Imaging examinations of 66 patients were classified by consensus as for the presence of anterior aorta displacement and were compared with the pathology report.

**Results:**

We found anterior abdominal aorta displacement in 26 (39.39%) of the 66 patients evaluated. Among those 26 patients, we identified neuroblastoma in 22 (84.62%), nephroblastoma in 3 (11.54%), and Burkitt lymphoma in 1 (3.85%). The positive predictive value was 84.62%, and the specificity was 88.24%. The displacement of the aorta was at the adrenal level, contralateral to the mass, in 14 cases, all of which were attributed to neuroblastoma.

**Conclusion:**

When the abdominal aorta is displaced at the level of the adrenal gland, contralateral to the mass, it can be said that the diagnosis is neuroblastoma, whereas abdominal aorta displacement occurring at other abdominal levels has a positive predictive value for neuroblastoma of approximately 85%.

## Abstract (pt)

**Objetivo:**

Determinar o valor do deslocamento tumoral anterior da aorta abdominal, quando presente em qualquer nível abdominal e quando presente somente ao nível da suprarrenal contralateral, na tomografia computadorizada e/ou ressonância magnética, para o diagnóstico de neuroblastoma, em crianças com até 7 anos de idade.

**Materiais e Métodos:**

Imagens de exames de 66 pacientes foram classificadas em consenso quanto à presença do deslocamento anterior da aorta e confrontadas com o anatomopatológico.

**Resultados:**

Observou-se deslocamento anterior da aorta abdominal, em qualquer nível abdominal, em 26 (39,39%) dos 66 pacientes. Desses 26 pacientes, 22 (84,62%) apresentaram neuroblastoma e os outros 4 (15,38%) representaram 3 casos de nefroblastomas e 1 de linfoma de Burkitt. O valor preditivo positivo foi 84,62% e a especificidade, 88,24%. Considerando o deslocamento da aorta somente presente quando ocorreu ao nível da suprarrenal contralateral à massa, observou-se que 14 casos eram neuroblastomas e nenhum outro tumor que não o neuroblastoma deslocava a aorta neste nível.

**Conclusão:**

Quando o deslocamento anterior da aorta abdominal é presente ao nível da suprarrenal contralateral à massa, pode-se afirmar que o diagnóstico é de neuroblastoma, ao passo que, quando presente nos demais níveis abdominais, sua capacidade em predizer neuroblastoma será de aproximadamente 85% nesta população.

## INTRODUCTION

Neuroblastoma, a malignant extracranial solid tumor that is most common among pediatric patients^(1)^, accounts for 7-10% of all childhood tumors, with a reported incidence of approximately 500 new cases per year in the United States^(2)^ . It also accounts for approximately 15% of all cancer-related deaths in the pediatric population^(3)^, its incidence peaking at 2-3 years of age^(4)^ . Cancer is the leading cause of death in children aged 1-14 years, accounting for 18% of all such deaths, and neuroblastoma is still the leading cause of cancer death in children and the third leading cause of death from malignant tumors in pediatric patients^(5)^ .

Neuroblastoma is the least differentiated of all neuroblastic tumors, showing an extremely variable biological behavior that can range from spontaneous regression to aggressive, disseminated disease^(6)^ . Neuroblastic tumors, which are derived from the neural crest of the sympathetic nervous system, are classified according to their degree of differentiation, as one of the following: ganglioneuroma, composed of ganglion cells and mature Schwann cells; ganglioneuro blastoma, composed of mature ganglion cells and immature neuroblasts with moderate malignant potential; or neuroblastoma, which is the most immature and least differentiated, with the greatest malignant potential^(7)^ . The most common presentation of neuroblastoma is in the retroperitoneum, 70% of all primary neuroblastomas being found in that region, most often in the adrenal glands or paravertebral ganglia. On physical examination, neuroblastoma can present as a palpable abdominal mass^(7)^ .

The variable biological characteristics of neuroblastomas, which can sometimes have severe, rapid development and a poor prognosis, lead us to seek simple ways in which to advance or guide the therapeutic support while awaiting the result of the biopsy. Characteristics frequently observed on axial computed tomography (CT) or magnetic resonance imaging (MRI) scans, which are often ordered for patients with abdominal masses, for which neuroblastoma is one of the differential diagnoses, allows us to predict the origin of the lesion, thus shortening the time to treatment.

In cases of neuroblastoma, involvement of the abdominal aorta has been reported since 1978, when Berger et al.^(8)^ described the first pediatric cases evaluated on the basis of axial CT images. Such findings were reported in greater detail in 1984 by Lowe et al.^(9)^, whose descriptions encompassed the vascular involvement in general, including that of the inferior vena cava, aorta, mesenteric vessels, and other vessels within the abdomen. Like other articles, the review published in 2002 by Lonergan et al.^(7)^ cited the involvement of vascular structures within the abdomen. However, none of the articles reviewed by those authors contained any mention of anterior displacement of the abdominal aorta by a neuroblastoma, which is a common finding on axial CT or MRI scans.

In 2011, Brisse et al.^(10)^ published protocols for the diagnostic imaging and staging of neuroblastic tumors, which included criteria for determining the radiological risks for neuroblastoma, as defined by the International Neuroblastoma Risk Group^(11,12)^, describing aortic entrapment as one such criterion.

Despite the extensive medical knowledge and the frequent descriptions of aortic involvement in neuroblastoma, there are no data available regarding the sensitivity and specificity of a finding of anterior displacement of the abdominal aorta on axial images and its ability to predict a diagnosis of neuroblastoma.

### Objectives

The objective of this study was to determine the sensitivity, specificity, positive predictive value (PPV), and negative predictive value (NPV) of anterior displacement of the aorta by a tumor, at any level of the abdominal aorta, on axial images obtained by CT or MRI.

An additional objective was to evaluate those same statistics when the displacement is observed only at the level of the adrenal gland contralateral to the mass, in the same series of CT or MRI images, seeking to establish the diagnosis of neuroblastoma.

We also attempted to determine whether there are differences related to the age of the patient and the size of the tumor in terms of the displacement or non-displacement of the abdominal aortic by a neuroblastoma.

## MATERIALS AND METHODS

This was a retrospective study that evaluated axial images of patients up to 7 years of age who had abdominal tumors seen on CT or MRI, with or without clinical suspicion of neuroblastoma, who were admitted to the hospital up through the end of 2013, and whose main complaint at admission was of an abdominal mass. Data were obtained from the hospital information system (HIS), and pretreatment images were obtained from the picture archiving and communications system (PACS) of the admitting facility.

A listing of the patients who fit into the design of the study was obtained from the hospital records. Inclusion and exclusion criteria were then applied. Patients who had no definitive pathology-confirmed clinical diagnosis were excluded, as were those for whom examinations were incomplete, those whom the evaluators considered technically inadequate for determining the position of the aorta, and those in whom no intra-abdominal tumor was identified during the review of the images.

The study was approved by the research ethics committee of the institution. After the inclusion and exclusion criteria had been applied, the hospital registry of eligible patients was encoded in ascending numerical order, the real hospital records maintained in anonymity to protect the identity of patients, and then sorted in descending order for the study. We then selected the initial axial CT or MRI images, available in the digital archives, obtained in any CT or MRI device, at any facility. The evaluators were blinded to data such as the final diagnosis, clinical findings, and reports of other tests.

**Figure 1:**
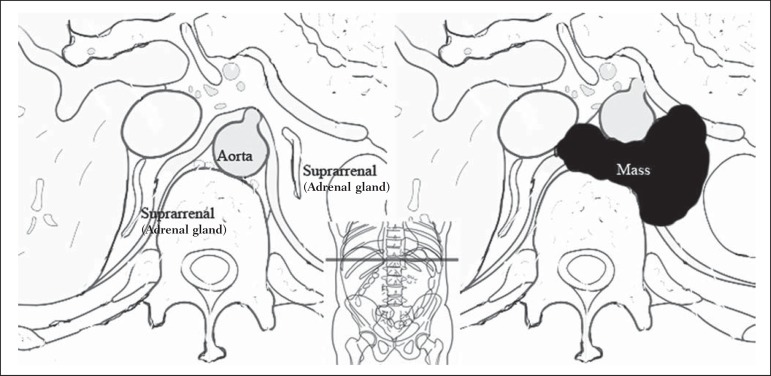
Schematic representation of the anterior displacement of the abdominal aorta at the level of adrenal gland on axial CT or MRI scans.

Two reviewers with expertise in pediatric radiology classified the CT or MRI scans by the presence or absence of anterior displacement of the abdominal aorta, or excluded patients based on the criteria mentioned above. Disagreements were resolved by consensus. The same images were again evaluated in order to identify cases in which there was anterior displacement of the abdominal aorta only at the level of the adrenal gland contralateral to the mass, as shown in Figure 1.

After the classification, we obtained the pathology results for the patients from the HIS and added them to the finding, categorizing the tests as neuroblastoma with displacement, neuroblastoma with no displacement, displacement with no neuroblastoma, or no neuroblastoma with no displacement. In a second analysis, the scans of the patients were again classified and we created a displacement group, comprising only those cases in which the displacement of the aorta was at the level of the adrenal gland contralateral to the mass. In the patients with pathology indicative of neuroblastoma, the abdominal masses were measured in an attempt to identify a correlation between the size of the neuroblastoma and the anterior displacement of the aorta. All of the data obtained were then tabulated and submitted to statistical analysis. The classification of the images by the evaluators was subjected to statistical analysis by Fisher's exact test to determine the significance of the findings. We then calculated the sensitivity, specificity, PPV, and NPV of anterior displacement of the abdominal aorta. Paired t-tests were used in order to evaluate the differences between mean age and neuroblastoma size. The level of significance was set at 0.05.

## RESULTS

A total of 87 patient electronic medical records in the HIS/PACS system of the facility were found to meet the requirements for inclusion in the study. Of those, 21 were excluded for being referred to the facility for post-treatment follow-up without initial images having been included in the PACS. Therefore, the study sample included 66 patients, and the sample calculation showed a moderate sample power (0.71). The mean age of the patients was 3.08 ± 1.92 years.

Anterior displacement of the abdominal aorta, at any level, was identified in 26 (39.39%) of the 66 patients. Among those 26 patients, a diagnosis of neuroblastoma was confirmed in 22 (84.62%). The remaining 4 patients (15.38%) had tumors other than neuroblastoma, 3 being diagnosed with Wilms tumor and 1 being diagnosed with Burkitt lymphoma. The finding of displacement of the abdominal aorta at any level presented a sensitivity of 68.75%, a specificity of 88.24%, a PPV of 84.62%, and a NPV of 75.0%. All these findings were statistically significant, at *p* < 0.001 (Table 1).

**Table 1:** Results of the statistical analysis of the correlation between anterior displacement of the abdominal aorta and a pathological diagnosis of neuroblastoma.

	Anterior displacement of the abdominal aorta		
	Value	Percentage	95% CI
Sensitivity*	0.6875	68.75%	0.49–0.83
Specificity*	0.8824	88.24%	0.71–0.96
PPV*	0.8462	84.62%	0.64–0.95
NPV*	0.75	75.00%	0.58–0.87
Accuracy	0.7878	78.78%	

* *P* < 0.001. PPV, positive predictive value; NPV, negative predictive value; 95% CI, 95% confidence interval.

**Figure 2:**
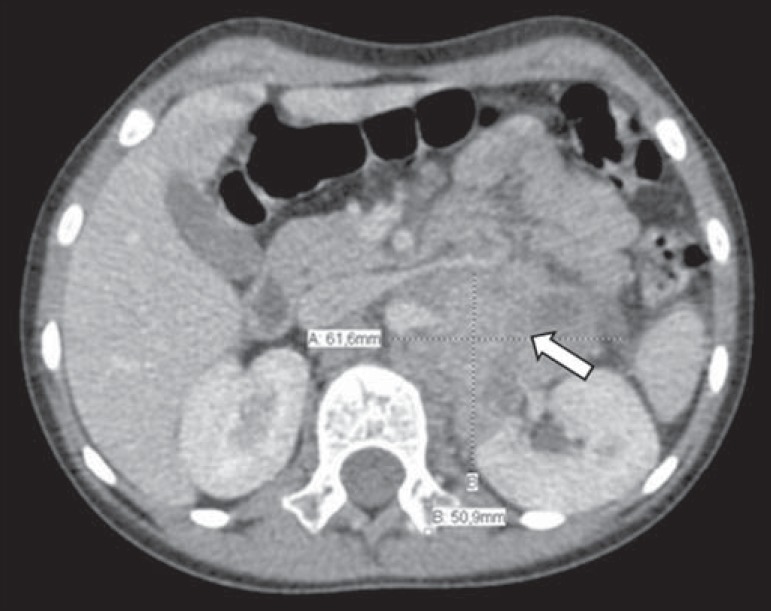
Axial CT image with intravenous contrast showing the anterior displacement of the abdominal aorta by a mass (arrow) that measured 6.16 cm at its greatest diameter.

**Figure 3:**
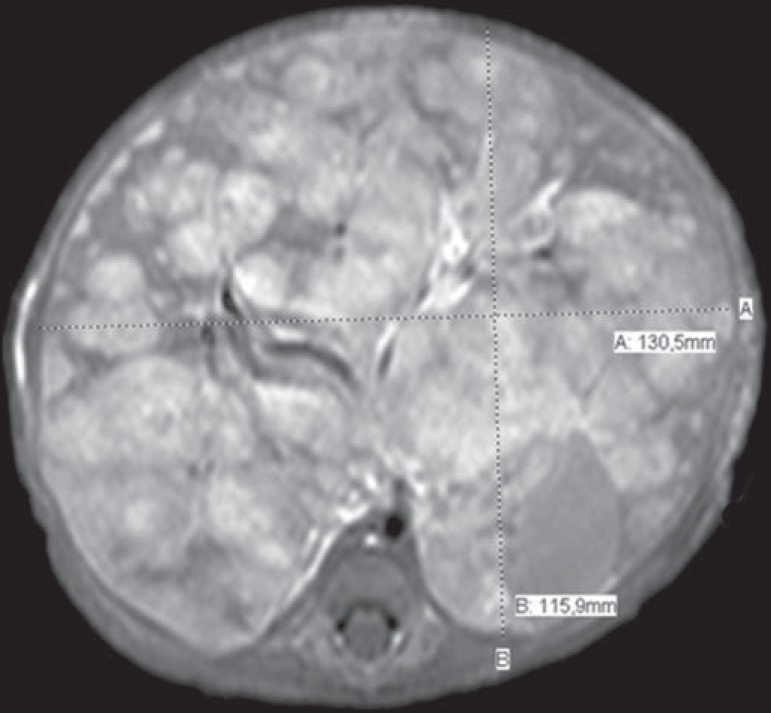
Axial T1-weighted MRI scan of a patient with a confirmed diagnosis of neuroblastoma that measured 13.05 cm at its greatest diameter and did not cause anterior displacement of the abdominal aorta.

Of the 66 patients evaluated, 32 (48.48%) had a confirmed diagnosis of neuroblastoma. Although those 32 patients had adrenal tumors that measured between 4.0 cm and 15.2 cm, 10 (31.25%) showed no anterior displacement of the abdominal aorta. The mean size of the neuroblastomas evaluated was 9.2 cm overall, being 9.66 ± 3.08 cm for those that displaced the aorta in the initial examination and 8.35 ± 3.45 cm for those that did not (Figures 2 and 3, respectively), with no statistically significant difference between the two (*p* = 0.289). The mean age of the patients was 3.14 years for those with neuroblastomas that displaced the aorta and 2.51 years for those with neuroblastomas that did not (*p* = 0.425).

**Figure 4:**
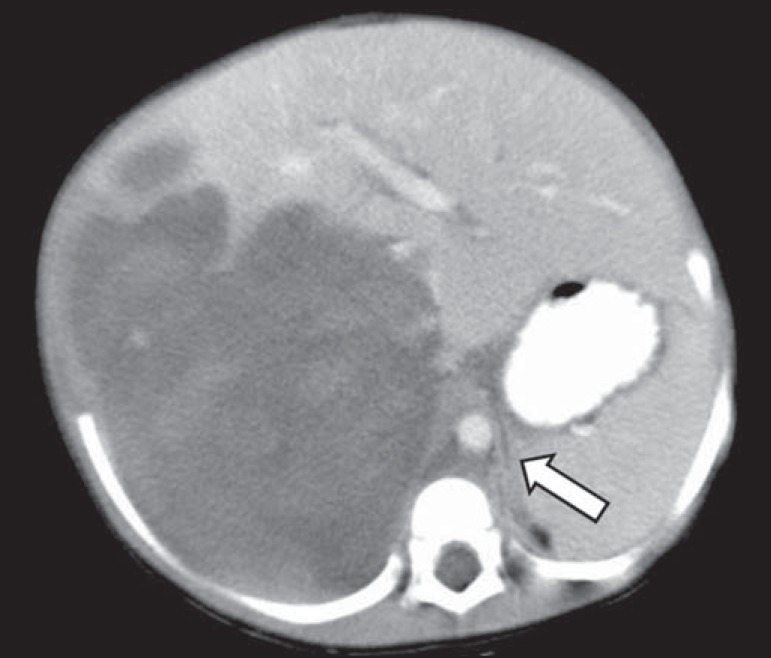
Axial CT scan with intravenous and oral contrast at the level of the adrenal gland contralateral to the mass (arrow), showing anterior displacement of the abdominal aorta and confirming the diagnosis of neuroblastoma

**Figure 5:**
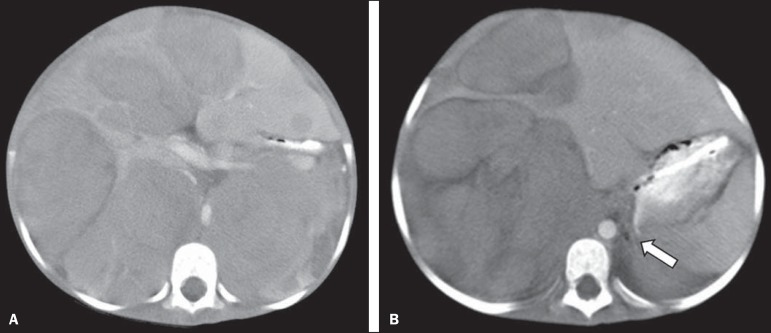
CT scan with intravenous and oral contrast of a patient with lymphoma who presented with anterior displacement of the aorta below the level of the adrenal gland (**A**), with no displacement at the level of adrenal gland (arrow, **B**).

**Figure 6:**
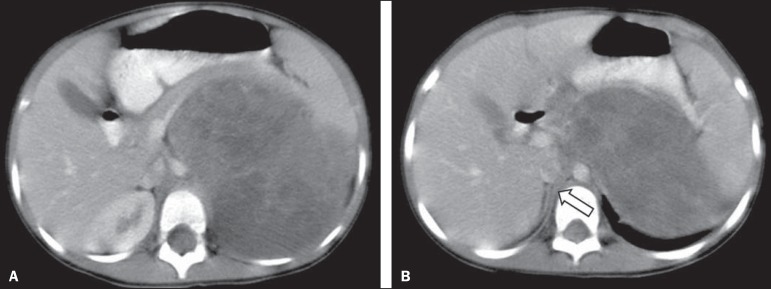
CT scans with intravenous and oral contrast of patients with masses that displaced the abdominal aorta. In the cases of Wilms tumor (**B**) there was no displacement of the aorta at the level of the adrenal gland (arrow) contralateral to the mass.

**Figure 7:**
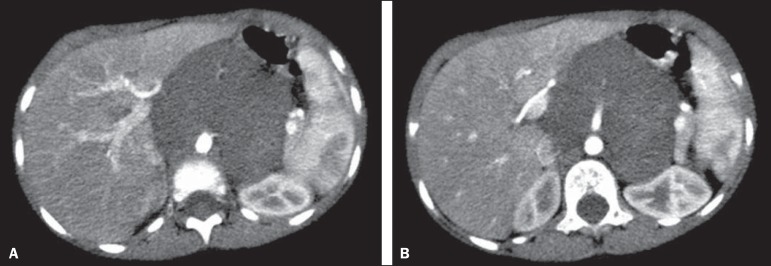
A: Axial CT scan with intravenous and oral contrast of a patient with a confirmed diagnosis of neuroblastoma, in whom there was no anterior displacement of the abdominal aorta at the level of the adrenal gland contralateral to the mass or at any other level, as in **B**.

Logistic regression showed that anterior displacement of the aorta at any level was a positive predictor of neuroblastoma (odds ratio: 16.5; 95% CI: 4.57-59.55; *p* < 0.001). In the 14 patients who showed displacement of the aorta at the level of the adrenal gland contralateral to the mass, the displacing tumors were neuroblastomas (Figure 4), and no tumor that was not a neuroblastoma displaced the aorta at that level (Figures 5 and 6). However, when we considered only that criterion, we found that the number of neuroblastomas that did not displace the aorta increased, from 10 to 18 (Figure 7), as did the number of non-neuroblastomas that did not displace the aorta, from 30 to 34.

There was also a significant increase in the specificity of the method (to 100%), together with a reduction in its sensitivity (to 43.75%), its PPV increasing (to 100%) and its NPV decreasing (to 65.38%). All of those findings were statistically significant, at p < 0.001 (Table 2).

Comparing the neuroblastomas that displaced the aorta with those that did not, we found that the size of the tumor made a statistically significant difference (*p* < 0.05). The mean size was 10.73 ± 2.37 cm for neuroblastomas that displaced the aorta, compared with 8.11 ± 3.35 cm for those that did not (*p* = 0.019). The mean age of the patients was 3.47 years for those with neuroblastomas that displaced the aorta and 2.53 years for those with neuroblastomas that did not (*p* = 0.192). Given the available number of patients in the sample, the sample calculation was carried out posteriorly, and the power of the sample was determined to be moderate.

**Table 2:** Results of the statistical analysis of the correlation between anterior displacement of the abdominal aorta at the level of the adrenal gland contralateral to the mass and a pathological diagnosis of neuroblastoma.

	Anterior displacement of the abdominal aorta		
	Value	Percentage	95% CI
Sensitivity*	0.4375	43.75%	0.36–0.61
Specificity*	1.0	100%	0.87–1.00
PPV*	1.0	100%	0.73–1.00
NPV*	0.6538	65.38%	0.58–0.87
Accuracy	0.7878	78.78%	

* *P* < 0.001. PPV, positive predictive value; NPV, negative predictive value; 95% CI, 95% confidence interval.

## DISCUSSION

Recent studies conducted in Brazil have highlighted the importance of imaging methods to improving diagnoses in the fields of pediatrics and neonatology^(13-20)^. The radiological aspects of neuroblastomas have been widely studied and described since the advent of axial CT and MRI scans. Despite the fact that anterior displacement of the abdominal aorta is a common finding in imaging studies of patients with neuroblastoma, a search of the literature revealed no statistical data related to that finding.

Imaging tests that suggest a diagnosis of neuroblastoma can be of extreme importance not only for the correlation with anatomic pathology findings but also to guide any type of urgent or emergency intervention by the clinical team while awaiting the final pathology report.

In the present study, all tumors that caused anterior displacement of the abdominal aorta at the level of the adrenal gland contralateral to the mass were identified as neuroblastomas in the pathology examination, the specificity and PPV of the finding both being 100%. However, not all neuroblastomas caused anterior displacement of the aorta. Some other cases of neuroblastomas that displaced the aorta at the lower levels might also not displace the aorta at the level of the adrenal gland when the tumor was at a slightly lower position.

Approximately 85% of the masses that caused anterior displacement of the abdominal aorta at any level were identified as neuroblastomas. Neither the size of the tumors nor the age of the patients had a statistically significant influence on the displacement of the aorta at any level. We noted that a neuroblastoma measuring only 6.16 cm displaced the aorta, whereas another that measured 13.05 cm did not.

Among the patients who showed displacement of the abdominal aorta at any level and did not have neuroblastoma, the conditions identified constituted the main differential diagnoses for the age group in question: Wilms tumor, in three cases; and Burkitt lymphoma, in one. None of those patients showed displacement of the aorta at the level of the adrenal gland contralateral to the mass.

Of the 32 patients with neuroblastoma, 10 (31.3%) showed no anterior displacement of the abdominal aorta at any level, demonstrating that not all neuroblastomas cause such displacement.

The calculated odds ratio allows us to state that anterior displacement of the abdominal aorta, at any level, results in a 16.5 times greater likelihood of a diagnosis of neuroblastoma, and that anterior displacement of the aorta at the level of the adrenal gland contralateral to the mass is diagnostic of neuroblastoma.

This study has some limitations. The fact that it was conducted at a referral center for pediatric oncology could have resulted in the de facto exclusion of many common, non-neoplastic, causes of abdominal masses, such as hydronephrosis. However, patients with such conditions would have subsequently been excluded for not presenting an abdominal tumor and therefore would have had no influence on the statistical analysis.

The available number of patients who met the study criteria could have limited our findings. Prospective multicenter studies, with interobserver analysis, aimed at consolidating this information in radiology practice could provide even more substantial data.

## CONCLUSIONS

We conclude that, when anterior displacement of the abdominal aorta is observed at the level of the adrenal gland contralateral to the mass, a diagnosis of neuroblastoma can be assumed, whereas, when the displacement of the aorta occurs at other levels, its ability to predict a diagnosis of neuroblastoma is approximately 85%, all the findings being statistically significant. It should be borne in mind that not all neuroblastomas cause anterior displacement of the abdominal aorta. Neuroblastomas not only have various clinical presentations but also have various presentations on imaging examinations.

Neither the size of the tumor nor the age of the patient appears to have any significant influence on whether a neuroblastoma causes anterior displacement of the aorta, although the size of the tumor appears to have a slight influence on displacement of the aorta at the level of the adrenal gland contralateral to the mass.
